# Four aspects to make science open “by design” and not as an after-thought

**DOI:** 10.1186/s13742-015-0072-7

**Published:** 2015-07-18

**Authors:** Yaroslav O. Halchenko, Michael Hanke

**Affiliations:** 1 Center for Cognitive Neuroscience, Dartmouth College, 3 Maynard Street, Hanover, 03755 NH USA; 2 Department of Psychology and Brain Sciences, 3 Maynard Street, Hanover, 03755 NH USA; 3 Debian Project, http://www.debian.org; 4 Psychoinformatics lab, Institute of Psychology II, Otto-von-Guericke-University, Magdeburg, Germany; 5 Center for Behavioral Brain Sciences, Magdeburg, Germany

**Keywords:** Neuroimaging, Open science, Intellectual property

## Abstract

Unrestricted dissemination of methodological developments in neuroimaging became the propelling force in advancing our understanding of brain function. However, despite such a rich legacy, it remains not uncommon to encounter software and datasets that are distributed under unnecessarily restricted terms, or that violate terms of third-party products (software or data). With this brief correspondence we would like to recapitulate four important aspects of scientific research practice, which should be taken into consideration as early as possible in the course of any project. Keeping these in check will help neuroimaging to stay at the forefront of the open science movement.

## Background

A long-standing relationship already exists between open science and neuroimaging research, primarily due to the fact that most research software in the field is free and open source software (FOSS). Many software toolkits for stimulus delivery and neuroimaging data processing were either developed as such from the beginning, or were relicensed under open-source licenses at some point. This rich collection prompted centralized software and data “clearing houses” such as the Neuroimaging Informatics Tools and Resources Clearinghouse (http://nitrc.org (NITRC)) [1, 2], and integrated turnkey software platforms such as the authors’ NeuroDebian (http://neuro.debian.net) [3, 4]. Increasingly, the software aspect of open science in neuroimaging is accompanied by open data, with public datasets being made available from archives such as OpenFMRI (http://openfmri.org) [5], the NITRC image repository (http://nitrc.org/ir (NITRC-IR)) [2, 6], and the Collaborative Research in Computational Neuroscience (http://crcns.org (CRCNS)) [7, 8] web portal. Despite these successes, incidents of neglected intellectual property (IP) norms, especially in scientific software, are not rare, even though neglecting or postponing IP issues poses a threat to a product’s (software or data) longevity and availability, and in turn the reproducibility of associated scientific results. For instance, the discovery of just a small, possibly even unused, snippet of code covered by a restrictive incompatible license can render *all* affected releases of a piece of software illegal, requiring their removal from public servers. A frequent example of this issue is the inclusion of example code shipped with the “Numerical Recipes” books (*e.g.,* [9]), in order to facilitate development by adoption of readily available implementations.

## Planning ahead

To enable future reproducibility, we first need to ensure the continued availability of today’s open science products. Therefore, we must be diligent in our compliance with established norms regulating IP, which are conversely the legal tool we can use to enforce persistent “openness”. We must make sure to obtain all necessary permissions to re-use or re-distribute third-party products and, in addition, determine under what conditions we can release our own work under open terms. It is important to understand that making your research products open to everyone *now* could be the only way to make them available to yourself in the *future*; for example, in case of a change of employment, or of a company policy. As it is impossible to provide an exhaustive advisory regarding IP laws, we will only outline the most important aspects, the first three of which concern both data and software projects, while the last one is mostly data-specific.

### Respect trademarks

Trademarks (commonly names and logos) exist to protect the identity of products or services and claim their exclusive properties. Trademark owners might pursue legal action if they find their trademark infringed upon, *e.g.,* if your related product has a similar name, or contains a trademarked name. Despite usually being resolved in private, we are aware of at least a few cases where authors of FOSS projects were contacted with *cease and desist* letters from corporations and were forced to pay fines for trademark infringement.

*Whenever deciding on a new project name or logo, verify that you are not infringing on an existing registered trademark, or in conflict with another open project. Both the US Patent and Trademark Office (*http://www.uspto.gov*(USPTO)) website and generic web search engines could be used to make a quick check. In the case of reusing names/logos of FOSS projects, check their trademark policies and consult the project owners.*

### Clarify ownership

The term *copyright* refers to the exclusive rights that may be enforced by some property owners. In the research context, there are typically three copyright-related issues to consider: 1) is a product *copyrightable*; and if so 2) who is the *owner*; and finally 3) do rights needs to be *transferred* to a third-party (*e.g.*, to a publisher)? Copyright applies to “any expressible form of an idea or information that is substantive and discrete” [10]. This also means that some materials may not be subject to copyright law. It is widely accepted that software (code and binaries), writing (articles, *etc.*), and artwork are copyrightable. The situation is less clear (and varies widely across different jurisdictions) in the case of application program interfaces (APIs) [see e.g., [11]] and data. For example, Creative Commons (CC) originally considered its license inappropriate for data [12], but this position was later rectified, recommending the data-oriented CC0 “no rights reserved” license [13], or the Public Domain Dedication and License (PDDL) [14], but also advising the use of CC licenses “where applicable/desired” [15, 16].

Generally authors hold the copyright of authored products, but if the product is a result of “work for hire”, the copyright is commonly either owned by the employer in some jurisdictions (*e.g.*, USA), or exclusively licensed to the employer where personal authors’ rights could not be transferred, as is the case in Germany [17]. It is common practice, then, that through the available legal norms, principal investigators sign off their rights to the work they were hired to do (often including off-work hours). Furthermore, rights to written works (*e.g.*, articles, books) are often transferred or exclusively licensed to a publisher, even for open access articles.

Limitations and exceptions to copyright [18], such as “fair use” in the USA [19] and “fair dealing” in the Commonwealth of Nations [20], exist to allow copyrighted works to be used without a license. However, their applicability is limited, varies widely across jurisdictions, and is open to interpretation, thus making reuse of those copyrighted works vulnerable to litigation.

*To guarantee perpetual open availability of your work it is first necessary to establish whether you could make it open. If unsure, make use of a “technology transfer” department or similar (e.g., a Copyright Specialist at the library and their online resources [e.g., [*21*]]). Clarify whether your product could be copyrighted, and who would own said copyright, given the details of the project funding and your status/contract. Be considerate when reusing any copyrighted materials. State the copyright (years, owner) for your copyrightable product and any third-party products you incorporate. When publishing, consider venues that do not require you to surrender your copyright or to provide exclusive rights.*

### Choose appropriate licenses

Licenses are tightly linked to the notion of copyright, defining rights granted by an IP’s owner that dictate how a product can be used and (re)distributed by a licensee. Moreover, many of the standard free and open source licenses include a disclaimer of any implicit warranty that could be associated with the product. Importantly, this is different from plain deposition of a product into the public domain (where applicable), as it may not provide this safety net.

The most common problem with licenses in the research context is related to the “borrowing” of source code from another product that was not released under a license permitting redistribution (as in the previously mention “Numerical Recipes” example) or imposing restrictions (*e.g.*, non-commercial use). The longer such incidents go unnoticed, the greater the negative impact for studies employing such products, and the greater the threat to the longevity of the product itself. A striking example of such a case is Astrolabe, Inc. vs. Olson et al. (tzdata database), in which Astrolabe claimed infringement by distributing factual data snippets copied from published atlases [22]. The authors of the tzdata database needed legal support from the Electronic Frontiers Foundation (EFF) to have the case dismissed. For sustainable open science we believe it is critical to release your work under a free and open license; it is just as critical to be pedantic in order to ensure the same freedom for all borrowed code and used products.

*If your institution/employer owns a product and the copyright, negotiate the choice of license with them. If work was performed as part of a grant submitted through your institution, chances are that an open license provision is already in place. Under all circumstances, avoid creating a custom license—use a standard one from Creative Commons (*http://creativecommons.org*) or Open Data Commons (*http://opendatacommons.org/licenses*), and ideally one that is known to conform to Debian Free Software Guidelines (*http://www.debian.org/social_contract#guidelines) [23*] and/or is Open Source Initiative (OSI) (*http://opensource.org/licenses*)-approved. License wording is non-trivial legalese; products with custom licenses are often neglected by third-party users because their legal implications are not fully understood. Do not impose additional (e.g., “no clinical use”) restrictions, unless unavoidable, to guarantee the widest possible adoption (see e.g., [*24*] for an analysis of common misconceptions about the conflict between open-source licenses and commercial interests). Choose a license appropriate to the product’s domain: software, web framework, documentation, artwork, data—they might require different licenses. Respect the licenses of the third-party products you use and make sure your license is compatible with their terms.*

### Obtain permission to share

Whenever products are shared, permission to do so must be given for all components with third-party rights. In general, this is implemented as a license. In neuroimaging research, there is one important special case: human subject data. For projects with human participants, protection of the participants’ privacy is of paramount importance when making imaging data publicly available. The respective norms are generally implemented as laws, such as [[25], 45 Code of Federal Regulations Part 46] in the US; adherence to these is scrutinized by institutional ethics committees, also known as institutional review boards (IRB). The decentralization of IRBs and the heterogeneity in their interpretation of the legal situation is one reason for the present lack of a *commonly accepted* language for participant consent forms to enable the sharing of research data. Consequently, many researchers simply exclude any data sharing statement in their consent forms to avoid frustration and delays in IRB evaluations. It is often neglected that the signed consent form is a document to protect researchers in the case that data has to be shared, for example, in order to comply with rules and regulations imposed by funding agencies, or publishers.

Although IRBs could warrant sharing of data previously collected without participants’ explicit agreement that their anonymized data may be publicly shared, it is in the experimenter’s interest to obtain explicit permission from participants to preclude any possible future legal trouble.

*Provision public data sharing via data archives in your consent forms before you begin collecting the data. The Open Brain Consent project (*http://open-brain-consent.readthedocs.org) [26] can be used to obtain samples of consent forms used at other institutions, and software for anonymization of data for sharing.

## Conclusion

Established norms behind intellectual property and participant privacy cannot simply be ignored if we would like to ensure the longevity of our open scientific projects. Due attention to the four aforementioned aspects from the beginning will reduce risks and foster sharing of methodologies, data, and results of your work later on—all activities inherent to “open science”.

## Abbreviations

API: Application Program Interface
CC: Creative Commons
CRCNS: Collaborative Research in Computational Neuroscience
EFF: Electronic Frontiers Foundation
FOSS: Free and Open Source Software
IP: Intellectual Property
IRB: Institutional Review Board
NITRC: Neuroimaging Informatics Tools and Resources Clearinghouse
NITRC-IR: Neuroimaging Informatics Tools and Resources Clearinghouse Image Repository
OSI: Open Source Initiative
PDDL: Public Domain Dedication and License
USTPO: United States Trademarks and Patents Office
